# Understanding the causal effect of migration on psychosis risk

**DOI:** 10.1192/j.eurpsy.2026.10336

**Published:** 2026-07-31

**Authors:** B. Della Rocca, J. Kirkbride, EU-GEI working group, PsyLife Group

**Affiliations:** 1 Psychiatry, University of Campania “L. Vanvitelli”, Naples, Italy; 2 Division of Psychiatry, UCL, London, United Kingdom

## Abstract

**Abstract:**

Migration is a well-established risk factor for psychotic disorders, yet the mechanisms underlying this association remain incompletely understood. Competing hypotheses have suggested either a selection effect, whereby individuals with increased vulnerability are more likely to migrate, or a causal role of migration-related exposures, including social adversity, discrimination, and environmental stressors. Distinguishing between these explanations is essential for understanding psychosis risk and identifying meaningful predictors of onset.

This presentation will discuss findings from the EU-GEI WP2 case–control study comparing first- and second-generation migrants and non-migrant groups with and without a first episode of psychosis. By accounting for underlying vulnerability while examining differences across migrant status and ancestry, the study evaluates whether migration-related risk can be explained by pre-existing susceptibility or whether environmental factors play a predominant role.

**Disclosure of Interest:**

None Declared

